# Imaging of Dopamine in PD and Implications for Motor and Neuropsychiatric Manifestations of PD

**DOI:** 10.3389/fneur.2013.00090

**Published:** 2013-07-09

**Authors:** Raúl de la Fuente-Fernández

**Affiliations:** ^1^Section of Neurology, Hospital A. Marcide, Complejo Hospitalario Universitario de Ferrol (CHUF), Ferrol, Spain

**Keywords:** dopamine, Parkinson’s disease, PET, motor function, fluctuations, dyskinesias, plasticity, neuropsychiatric manifestations

## Abstract

Parkinson’s disease (PD) is characterized by dopamine depletion in the putamen, which leads to motor dysfunction. As the disease progresses, a substantial degree of dopamine depletion also occurs in caudate and nucleus accumbens. This may explain a number of neuropsychiatric manifestations, including depression, apathy, and cognitive decline. Dopamine replacement therapy partially restores motor function but long-term treatment is often associated with motor complications (motor fluctuations and dyskinesias). Positron emission tomography (PET) studies suggest that the dopamine release rate is substantially higher in PD subjects with motor complications compared to stable responders. Notably, this differential pattern of dopamine release is already present in the early stages of the disease, before motor complications become clinically apparent. Converging evidence suggests that striatal dopamine depletion in PD leads to reduced plasticity in the primary motor cortex and, presumably, in non-motor cortical areas as well. Although dopamine replacement therapy tends to restore physiological plasticity, treatment-induced motor, and neuropsychiatric complications could be related to abnormalities in corticostriatal synaptic plasticity.

## Introduction

Parkinson’s disease (PD) has traditionally been defined according to its motor manifestations. Bradykinesia, rigidity, and resting tremor are the core clinical features and reflect the degree of dopamine depletion in the putamen (1, 2). Recent years have seen an increasing interest in neuropsychiatric manifestations of the disease, some of which are likely related to dopamine depletion in the caudate nucleus and nucleus accumbens (NAcc). In fact, a putamen-caudate-accumbens gradient of dopamine depletion, with dysfunction of the corresponding frontostriatal loops, followed by dopamine depletion in the frontal cortex, has been proposed to explain the sequential occurrence of motor symptoms and a great variety of neuropsychiatric manifestations (3). In addition to the clinical picture observed during the “off” medication state, there are also treatment-related motor and non-motor alterations. Dopaminergic (DA) therapies are able to normalize motor function and may also help to correct neuropsychiatric symptoms during the early stages of the disease. In the long-term, however, these therapies are often associated with motor and non-motor complications. Dyskinesias and impulse control disorders (ICDs) are only two examples. There is compelling evidence to suggest that dyskinesias are due to treatment-related abnormalities occurring in the frontostriatal motor loop (4, 5). Likewise, treatment-related abnormalities in the frontostriatal cognitive and limbic loops could be responsible for ICDs. This short review article will cover some of the most relevant contributions of dopamine radiotracer neuroimaging to the understanding of motor and neuropsychiatric manifestations of PD.

## PET Assessments of Dopaminergic Function

A number of positron emission tomography (PET) radiotracers can be used to assess the DA system. Presynaptically (6), (1) [^18^F]-fluorodopa uptake provides an estimate of the activity of the enzyme dopa-decarboxylase, (2) plasma membrane dopamine transporter (DAT) radioligands (e.g., [^11^C]-methylphenidate) provide an estimate of DAT site density, and (3) vesicular monoamine transporter type 2 (VMAT2) radioligands (e.g., [^11^C]-dihydrotetrabenazine) provide an estimate of VMAT2 site density. Postsynaptically, [^11^C]-raclopride (a D2/D3 receptor antagonist) is the radioligand most frequently used to estimate the density of dopamine D2/D3 receptors in the striatum, where receptor concentrations are high. For extrastriatal areas (e.g., frontal cortex), where D2/D3 receptor concentrations are low, raclopride does not provide an optimal signal-to-noise ratio. Here, high-affinity D2/D3 antagonists such as [^18^F]-fallypride and [^11^C]-FLB-457 are better biomarkers (7, 8). A major advantage of raclopride is its susceptibility to displacement by dopamine (5, 9). This allows comparisons between baseline and postactivation scans in order to estimate the amount of dopamine released after the activation of the DA system (e.g., after levodopa challenge). In contrast, the ability of high-affinity radiotracers to quantify dopamine release in extrastriatal areas seems to be limited.

## Degeneration of dopamine pathways in PD

Post-mortem biochemical studies have shown that PD has a characteristic gradient of dopamine depletion in the striatum (1, 2). The putamen is the most affected region, followed by dorsal caudate (d-Caud), ventral caudate (v-Caud), and NAcc. Longitudinal PET studies have confirmed *in vivo* such a progressive putamen-caudate gradient of DA dysfunction (10), although specific subregions of the ventral striatum were not evaluated. In all likelihood, this gradient of dopamine depletion reflects the sequential degeneration of the nigrostriatal and mesolimbic dopamine pathways. Presumably, the mesocortical dopamine pathway would be affected last, which would lead to cortical dopamine depletion.

Based on the striatal gradient of dopamine depletion, three major anatomical and functional frontostriatal loops are predicted to be sequentially affected in PD (3): first, the motor loop, which connects cortical motor areas (including the supplementary motor cortex and the primary motor cortex) with the putamen; second, the cognitive loop, which connects the dorsolateral prefrontal cortex (DLPFC) with the dorsal caudate nucleus (d-Caud); and third, a “complex” limbic loop, with connections (i) between the orbitofrontal cortex (OFC) and the v-Caud nucleus (v-Caud), and (ii) between the anterior cingulate cortex (ACC) and the NAcc (11– 14). Over time, these frontostriatal loop dysfunctions caused by dopamine depletion in the striatum would be further complicated by cortical dopamine depletion secondary to degeneration of the mesocortical dopamine pathway – the direct DA projection to the frontal cortex. The degree of frontal DA dysfunction might follow a gradient similar to that proposed for the striatum (i.e., DLPFC > OFC > ACC) (15). Dopamine neurons of the mesocortical pathway have unconventional characteristics, including lack of DAT and D2 autoreceptors (16), which may limit their capability to undergo regulatory adaptations.

## Dopamine and Motor Manifestations of PD

A distinction should be made between DA dysfunction “off” and “on” medication.

### Striatal dopaminergic dysfunction during the “off” medication state

**Figure 1:**
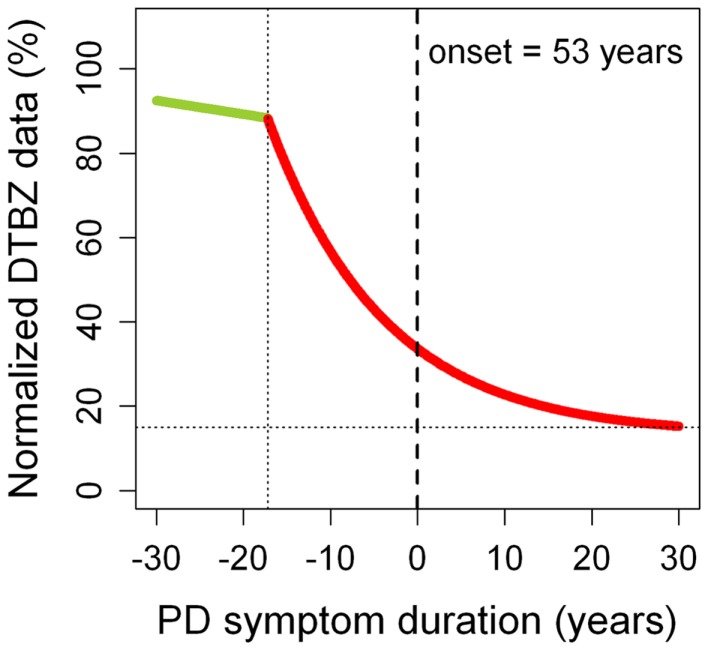
**Progression of putaminal dopaminergic (DA) dysfunction in Parkinson’s disease (PD)**. DA dysfunction was estimated using normalized [^11^C]dihydrotetrabenazine (DTBZ) binding data. The green straight segment represents normal aging. The red curve corresponds to PD. Motor symptoms begin at time 0 (in this case, at age 53 years, which was the mean age of PD onset of the sample). A very similar PD curve was obtained for the caudate nucleus (10). These observations suggest that the nigrostriatal and mesolimbic dopamine pathways probably have the same pattern of neurodegeneration. It remains unknown whether the same applies to the mesocortical dopamine pathway. Adapted from Ref. (17).

A recent multi-tracer longitudinal PET study has demonstrated *in vivo* that DA dysfunction in PD is particularly severe in the putamen (17). Using a VMAT2 marker ([^11^C]dihydrotetrabenazine – DTBZ), it was estimated that some 70% of DA terminals must be lost before the first motor symptoms occur (Figure 1), which agrees with post-mortem estimates of striatal dopamine depletion (80%) (1, 2). Different radiotracers offer, however, somewhat different pictures (6). Thus, DAT radioligands tend to give more severe estimates of DA dysfunction, probably reflecting compensatory downregulation of DAT sites in an attempt at maintaining normal synaptic dopamine levels. In contrast, fluorodopa uptake is relatively upregulated in PD, which may also represent a compensatory mechanism. In consequence, there is a consensus that VMAT2 radioligands probably provide more accurate estimates of DA dysfunction (6), although VMAT2 binding may be also subject to some degree of regulation (18). In general, bradykinesia and rigidity scores (but not tremor scores) correlate with the nigrostriatal DA deficit (19, 20). Imaging markers of DA dysfunction may not distinguish between idiopathic and monogenic parkinsonism (21).

Putaminal DTBZ binding curves obtained from longitudinal PET data have provided important information on the progression of PD. It has been estimated that the onset of DA pathology (loss of DA terminals in the striatum) begins some 17 years before the first motor symptoms appear (Figure 1). Notably, this presymptomatic phase of the disease is longer in younger patients (25 years) compared to older patients (10 years). Once motor symptoms begin, there seems to be little progression after about 5–10 years into the symptomatic phase of the disease. In fact, about 15% of putaminal DA terminals still remain intact in very advanced PD stages (i.e.,>30 years after motor symptom onset). These observations suggest that a substantial part of the progression in motor dysfunction that we see in our patients in the clinic may not reflect the progressive loss of DA terminals, but maladaptive changes occurring in dopamine-dependent frontostriatal loops. For example, a number of studies support the view that cortico-cerebello-thalamo-cortical loops increase activity to compensate for dysfunctional changes occurring in the cortico-striato-thalamo-cortical loops, in an attempt to maintain near-normal motor function (22). In the long-run, however, these initially beneficial compensations may end up contributing to the development of motor complications (23). In addition, PD-related pathological changes (i.e., alpha-synuclein deposition) occurring in non-DA systems can also contribute to the clinical progression.

### Striatal Dopaminergic Dysfunction During the “On” Medication State

Dynamic PET studies with raclopride have revealed a number of adaptations that occur in surviving DA terminals during the symptomatic phase of PD. Among all the mechanisms involved in the dopamine release-reuptake cycle, alterations of a parameter, namely the dopamine release rate, has been identified as the most important risk factor for the development of treatment-related motor complications (4). Specifically, patients with early PD who will go on to develop motor complications (fluctuations and dyskinesias) have a higher dopamine release rate than those who will remain stable responders (9). This between-group difference increases over time, being particularly prominent once motor complications become clinically relevant (5). At that time, fluctuators and dyskinetics release very large amounts of dopamine during the first hour after oral administration of levodopa. The abnormal increase in levodopa-related dopamine release will lead to (i) dyskinesias (reflecting large swings in synaptic dopamine levels), (ii) fluctuations (whenever the dopamine reuptake system is not efficient enough to maintain adequate presynaptic dopamine levels for the following release-reuptake cycles), or (iii) a combination of the two. In contrast, stable responders show a more physiologic and sustained dopamine release process, maintaining adequate synaptic dopamine levels during hours following levodopa challenge. It should be emphasized that although PD patients with motor complications have an increase in the release of dopamine during the first hour after levodopa administration, it does not mean that the overall synaptic levels of dopamine reach above normal levels in PD. Levodopa treatment does normalize vesicular dopamine levels in surviving striatal DA terminals but the overall synaptic levels of dopamine remain below normal due to the profound loss of striatal DA terminals (4, 5). In keeping with this notion, dyskinetics often have residual parkinsonism during the “on” medication state.

Interestingly, neurophysiologic studies using transcranial magnetic stimulation methods show that the differential pattern of putaminal dopamine release (and the corresponding differential pattern of putaminal dopamine receptor stimulation) observed in patients with motor complications and stable responders has a correlate in the frontostriatal motor loop (24). Thus, while levodopa treatment is able to restore normal plasticity in the primary motor cortex of stable responders, primary motor cortex plasticity dramatically declines in patients with motor complications. As there is some indication that differential patterns of dopamine release may also occur in other striatal structures (caudate and accumbens), the possibility exists that plasticity changes occurring in the corresponding frontostriatal loops (i.e., cognitive and limbic loops) could play a role in the pathogenesis of a number of neuropsychiatric manifestations of PD. For example, ICDs could be related to a relative overactivity of the mesolimbic dopamine pathway (12, 25), in the same manner as dyskinesias are related to a relative overactivity of the nigrostriatal dopamine pathway.

## Dopamine and Neuropsychiatric Manifestations of PD

### Model of neuropsychiatric manifestations

**Figure 2:**
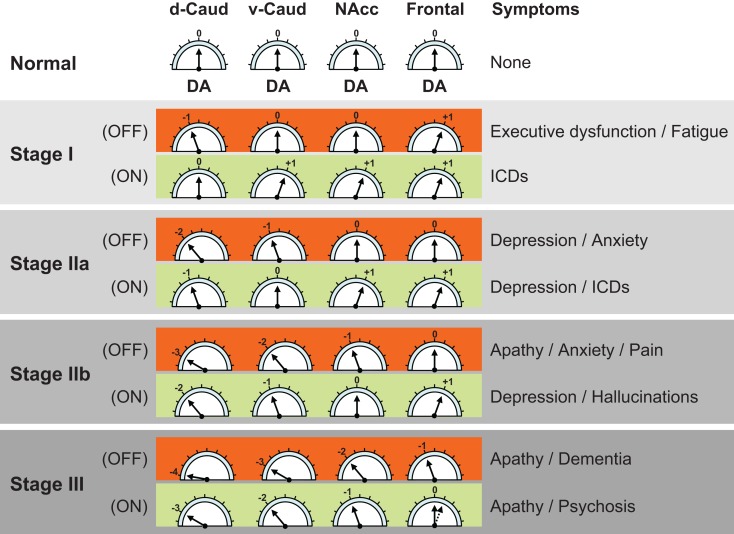
**Parkinson’s disease related frontostriatal cognitive dysfunction (PDFCD) staging with region-specific dopaminergic (DA) tone**. Predicted stage-specific DA function, both “off” and “on” medication (OFF and ON), is shown for dorsal caudate (d-Caud), ventral caudate (v-Caud), nucleus accumbens (NAcc), and frontal cortex. Although no distinction is made between the frontal regions corresponding to the different frontostriatal cognitive loops (i.e., dorsolateral prefrontal cortex, orbitofrontal cortex, and anterior cingulate cortex), a gradient of DA dysfunction may be also present in the mesocortical dopamine pathway (15). The DA tone can be optimal (0), too low (negative values) or too high (positive values). The direct DA projection to the frontal cortex seems to be initially upregulated (29), but with limited capability to increase further the DA tone in response to DA treatment because it lacks dopamine transporter sites and dopamine D2 autoreceptors (16). ICDs = impulse control disorders. Adapted from Ref. (3).

A recently reported PD-related frontostriatal cognitive dysfunction (PDFCD) staging model suggests that a number of neuropsychiatric manifestations of the disease may follow a staging of DA dysfunction in frontostriatal loops (3) (Figure 2). Accordingly, fatigue, depression (OFF), and apathy would occur sequentially in the “off” medication state. Likewise, ICDs, depression (ON), and psychosis would be the corresponding neuropsychiatric manifestations during the “on” medication state. ICDs, in particular, are proposed to be due to a relatively excessive DA drive in non-motor frontostriatal loops, and could therefore be the neuropsychiatric equivalent to dyskinesias.

The PDFCD stating is fundamentally based on the DA tone present in different striatal structures (d-Caud, v-Caud, and NAcc), and is supported by the sequential abnormalities observed in tests that are specific for different frontostriatal loops (12, 25–28). The contribution of the direct mesocortical DA projection to loop-specific cortical areas (i.e., DLPFC, OFC, and ACC) remains unclear, although fluorodopa PET studies suggest that the DA tone in the frontal cortex may be upregulated in early PD (29). Whether such, presumably compensatory, frontal DA upregulation is region-specific for dysfunctional frontostriatal loops remains unknown. Over time, however, frontal DA overactivity is expected to disappear as the mesocortical dopamine pathway becomes affected by PD pathology. Eventually, these dopamine-related striatal and cortical functional alterations of the frontostriatal loops would become complicated by the addition of cortical PD pathology (i.e., alpha-synuclein deposition in the frontal cortex).

#### Impulse control disorders

Impulse control disorders in PD are a group of neuropsychiatric manifestations associated with younger age, treatment with dopamine agonists, and use of high doses of levodopa (30, 31). The estimated prevalence is approximately 15%. Pathological gambling, compulsive shopping, compulsive eating, and hypersexuality are the most frequently reported ICDs. Compulsive DA medication use, particularly levodopa abuse, is a related disorder typically associated with younger age and depression. Punding (excessive, unproductive repetitive motor actions) is also a compulsive behavior sometimes encountered in PD. For simplicity, all these abnormal behaviors can be considered under the umbrella of ICDs, although the hedonic component may vary considerably among them (e.g., it would be large in hypersexuality and relatively low in punding).

The PDFCD model suggests that dysfunction of specific frontostriatal loops plays a pivotal role in ICDs (Figure 2). Excessive striatal DA activation of the loops connecting the v-Caud nucleus with the OFC (v-Caud-OFC loop) and the NAcc with the ACC (NAcc-ACC loop) has been implicated in the pathogenesis of pathological gambling (32) and compulsive DA medication use (33). These observations are in keeping with the reported increase in amphetamine-induced release of dopamine in the striatum of non-PD impulsive individuals, as estimated by [^18^F]-fallypride binding changes (34). Interestingly, while raclopride PET studies show that pathological gambling is associated with an increase in the release of dopamine in the ventral striatum (32), dopamine D2/D3 receptor availability (as estimated by [^11^C]-FLB-457 PET) is increased in the ACC (35), suggesting that the mesocortical dopamine pathway may indeed be underactive. ICDs could therefore be associated with increased DA tone in the ventral striatum and decreased DA tone in the ACC. As mentioned earlier, evidence from fluorodopa PET studies suggest that the mesocortical dopamine pathway is overactive in early PD (29), perhaps as a compensatory mechanism for striatal dopamine depletion. Hence, the DA underactivity observed in the ACC of PD patients with pathological gambling could be also compensatory for the excess of DA tone in the ventral striatum.

#### Depression and apathy

In contrast to PD patients with pathological gambling, who have altered striatal and cortical DA homeostasis, with increased DA tone in the ventral striatum and low DA tone in the ACC (35), PD patients with apathy have reduced DA tone in both striatum (ventral and dorsal striatum) and prefrontal cortex (36). Thus, as predicted by the PDFCD model, apathy is associated with low DA tone in the dorsolateral prefrontal, orbitofrontal, and ACC loops (Figure 2). The model also predicts that depression and anxiety are clinical predictors of apathy and may also reflect DA dysfunction. In fact, mood and anxiety fluctuations sometimes mirror DA tone changes in PD patients with motor fluctuations (37, 38), although “on” medication depression can also occur. There is some evidence that dopamine function may be also altered in non-PD individuals with depression (39, 40). This may be particularly true of melancholic depression, which is typically characterized by anhedonia (41). Naturally, other DA and non-DA factors may also contribute to depression and anxiety in PD. The amygdala, for example, which is known to play an important role in anxiety disorders (42), can undergo DA denervation as well as direct PD-related pathological changes (43, 44).

#### Psychosis

The development of psychosis represents a later PDFCD stage, where DA dysfunction affects not only the striatum (dorsal and ventral striatum) but also the direct DA projection to the frontal cortex (i.e., the mesocortical dopamine pathway) (Figure 2). Clinically, psychosis is often preceded by relatively benign visual hallucinations, signaling the involvement of the NAcc – ACC loop. By the time psychosis occurs, the “off” medication DA tone is predicted to be low at both the striatal and cortical levels. It is tempting to establish some conceptual parallelism between PD-psychosis and schizophrenia. Converging evidence suggests that schizophrenia is characterized by a hyperdopaminergic tone in the ventral striatum associated with a hypodopaminergic tone in the frontal cortex (45). The PDFCD model, on the other hand, suggests that a widespread hypodopaminergic tone in both striatal and cortical areas, even during the “on” medication state, may characterize psychosis in PD. Nevertheless, hallucinations and psychosis in PD could be due to large fluctuations in the level of DA stimulation occurring during the “on” medication state rather than an overall hypo- or hyperdopaminergic tone. This raises the possibility that some frontostriatal loops may be relatively overactive during the “on” medication state, which would explain why, as with dyskinesias, decreasing the dose of DA drugs is often helpful for ameliorating hallucinations and psychosis. Taking into account that the DA tone in the frontal cortex is mostly determined by dopamine D1 receptors (46–48) and that hallucinations and psychosis are especially associated with the use direct dopamine D2 agonists (49, 50), it seems reasonable to conclude that PD-psychosis is likely associated with a relative hyperdopaminergic D2 tone in the NAcc. Interestingly, [^11^C]-FLB-457 binding in the ACC decreases as PD progresses (15), which could suggest a compensatory increase in the activity of the direct DA projection to that frontal area. In addition to striatal and cortical DA dysfunction, cortical alpha-synuclein pathology can be a major determinant for the onset of psychosis.

## Concluding Remarks

Dopamine radiotracer neuroimaging has greatly contributed to increase our understanding of motor and neuropsychiatric manifestations of PD. It is becoming increasingly clear that many of the clinical manifestations observed during the “on” state are not the result of an average hyperdopaminergic tone, but are due to the presence of large swings in synaptic dopamine levels. This mechanism explains dyskinesias and may also apply to ICDs and psychosis. Further research is needed to better characterize the progression of DA dysfunction and plasticity changes in different frontal regions and the corresponding clinical correlates.

## Conflict of Interest Statement

The authors declare that the research was conducted in the absence of any commercial or financial relationships that could be construed as a potential conflict of interest.
